# The Journey to Autonomy: Understanding Parental Concerns During the Transition of Children with Chronic Digestive Disorders

**DOI:** 10.3390/medicina61081338

**Published:** 2025-07-24

**Authors:** Silvia Cristina Poamaneagra, Sorin Axinte, Carmen Anton, Elena Tătăranu, Catalina Mihai, Gheorghe G. Balan, Georgiana-Emmanuela Gîlca-Blanariu, Oana Timofte, Frenți Adina Mihaela, Oana Maria Roșu, Liliana Anchidin-Norocel, Smaranda Diaconescu

**Affiliations:** 1 Doctoral School, George Emil Palade University of Medicine, Pharmacy, Science and Technology, 540139 Târgu Mureș, Romania; silviastrat89@yahoo.ro (S.C.P.); savu.adina-mihaela.24@stud.umfst.ro (F.A.M.); 2 Faculty of Medicine and Biological Sciences, Stefan cel Mare University of Suceava, 720229 Suceava, Romania; etataranu@gmail.com (E.T.); liliana.norocel@usm.ro (L.A.-N.); 3 Clinical Department of ENT, Sf. Ioan cel Nou, Emergency Hospital, 720237 Suceava, Romania; 4 Faculty of Medicine, “Grigore T. Popa” University of Medicine and Pharmacy, 700115 Iasi, Romania; catalinamihai@yahoo.com (C.M.); balan.gheo@me.com (G.G.B.); georgiana.gilca@gmail.com (G.-E.G.-B.); oana.timofte@umfiasi.ro (O.T.); 5 Department of Gastroenterology and Hepatology, “St. Spiridon” Emergency Hospital, 700115 Iasi, Romania; 6 Clinical Department of Pediatrics, Sf. Ioan cel Nou, Emergency Hospital, 720237 Suceava, Romania; 7 Clinical Department of Neonatology, Sf. Ioan cel Nou, Emergency Hospital, 720237 Suceava, Romania; 8 Clinical Department of Pediatric Gastroenterology, Elytis Hospital, 700010 Iasi, Romania; rosuoanamaria@elytis-hospital.ro; 9 Faculty of Medicine, “Titu Maiorescu” University of Medicine, 040441 Bucharest, Romania; smaranda.diaconescu@prof.utm.ro

**Keywords:** transition, chronic digestive disease, parental involvement, parents’ fears, parents’ wishes, adolescent independence, health decision-making, continuity of care

## Abstract

*Background and Objectives*: The transition from pediatric to adult-oriented healthcare is challenging and data on parental involvement and perception regarding the transition of children with chronic digestive diseases are scarce. *Materials and Methods*: Legal guardians of adolescents with chronic digestive diseases receiving care at a North-Eastern Romanian tertiary center and private offices were administered a 30-item survey. *Results*: There were 124 responders; 73.4% lived in rural areas; 81.5% were patients’ mothers. Positive correlations were found between parents’ perception of the child’s readiness for health-related decisions and appreciation of the children’s preparedness for transition (0.544; *p* = 0.000), between parents encouraging their children to maintain healthcare records and their perception of the children’s knowledge about their disease (0.67; *p* = 0.000), between parents’ fear of therapeutic breaks during transition and their perception of the need for transition training (0.704; *p* = 0.000), between fears for children’s impropriate health-related choices, fears of therapeutic breaks (0.573; *p* = 0.00) and parental perception that the adult physicians would be more patient-oriented and less family-centered (0.453; *p* < 0.000) and between parents’ trust in their children’s self-management skills and encouraging them to make decisions on their own (0.673; *p* < 0.000). *Conclusions*: The results of our study highlight the importance of addressing parental fears during special parent–children counseling sessions and promoting a child’s independence, chronic disease knowledge, records and alone consultations.

## 1. Introduction

The transition from adolescence to adulthood is a critical period, particularly for adolescents managing chronic health conditions, as it involves not only the shift from pediatric to adult healthcare services but also the increasing need for self-management and independence. Parental involvement plays a pivotal role during this transition, as parents often act as primary caregivers and advocate for their children’s health needs. However, the process of “letting go” can be fraught with emotional challenges and practical concerns for parents [1].

Transition refers to the complex process of addressing the medical, psychosocial and educational needs of chronically ill adolescents as they prepare to move toward adult-centered healthcare. Transfer, on the other hand, represents the physical change in papers, responsibility, location and healthcare provider [2].

Transfer of responsibility is neither linear nor straightforward for family members; parents, in particular, often struggle to determine what is ‘normal’ and how much autonomy to allow [3].

This period is particularly critical, with increased parental stress and a heightened risk of depression if independence does not develop or if coping skills are inadequate. Parents frequently experience tension between the need to protect their child and the desire to promote autonomy [4,5].

The literature identifies three major needs of patients and their caretakers in the pre-transfer period. In the first place, there is the need to anticipate the change in the medical team, and, while choosing the institution responsible for the subsequent management of the patient, future plans and mobility of the patient and/or their family must be taken into account. Afterwards, it is imperative to understand the principles of self-management of the disease. An essential role in achieving this goal is played by the legal guardians who should understand this need, and should offer support and freedom to the future young adults. All these principles highlight the concept already stated by the specialized literature according to which the transition process should be a smooth organized one, rather than a rushed transition based on a simple transfer that could put the entire process at risk of failure [6].

The primary role of the family remains of paramount importance in the development of self-care abilities, in informing and supporting the young adult and in promoting and encouraging healthy behaviors regarding the chronic condition. However, parental involvement in this process is difficult to quantify on a daily basis and the implications of a malfunctioning home environment could be catastrophic for a teenager suffering from a chronic disease.

The needs of parents have been largely overlooked, creating a significant gap in research on how these parents manage their child’s autonomy acquisition and the factors that influence their encouragement or discouragement of independence [7,8].

Recent data estimate that up to 40% of adolescents lose access to healthcare during the transition process from pediatric to adult medical care, leading to increased rates of complications and decreased quality of life [9].

In order to avoid such negative consequences, structured and planned transition protocols have been proposed by specialized fora from different countries [10,11,12,13,14].

In Romania, there is no formal protocol for the transition of adolescent patients with chronic diseases, and no published data regarding the main actors involved in the process: patients, their families and healthcare professionals.

Therefore, one important part of translational care is to understand the way parents experience their children’s process of transition to adulthood because the manner in which they cope with their own challenges and responsibilities will impact on their children’s transfer to independent self-care and on their psychological well-being [15].

The goals of this study are the following:

➢ To realize a sociodemographic characterization of the studied population;
➢ To establish the particularities of families and children with chronic digestive diseases living in the North East Region of Romania, who are on the verge of transitioning from the pediatric to the adult-oriented healthcare system;
➢ To identify parental appreciation of their children’s abilities of self-management of the chronic disease;
➢ To identify parents’ perception related to the transition process;
➢ To identify parents’ feelings, needs and worries related to the transition process;
➢ To identify parents’ wishes related to the transition;
➢ To identify potentially modifiable parent-related risk factors that could be addressed through active interventions.

## 2. Materials and Methods

### 2.1. Participants and Sampling

To address the study objectives, we conducted a cross-sectional survey. We enrolled a total of 124 legal guardians of patients treated for a chronic digestive condition at the Pediatric Gastroenterology Clinic from the Clinical Emergency Children’s Hospital St. Mary from Iasi, Romania, and private practice offices. The inclusion criteria consisted of the following: caring for at least one child with a chronic digestive disease defined according to the ESPGHAN (European Society for Pediatric Gastroenterology Hepatology and Nutrition) criteria in the records of a doctor in the clinic, the child’s age being between 14 and 17 years and signing the informed consent for participation in the study. Patients were treated for inflammatory bowel diseases (ulcerative colitis and Crohn’s disease), chronic gastritis, celiac disease and chronic liver diseases (chronic hepatitis B, chronic hepatitis C, cystic fibrosis with hepatic involvement, liver cirrhosis, Pompe disease with hepatic involvement).

The study was approved by decision no. 38761/09.11.2020 of the Research and Ethics Commission of “St. Maria Hospital”.

### 2.2. Survey Design and Validation

The questionnaire was created by our research team and consisted of 30 clearly formulated closed questions and could be completed within 5 min. Because it was developed for caregivers with varying levels of formal education, the items were both relevant to the investigation and easy to understand. The questions targeted the main interested domains that could influence the patient’s transition process, such as family components, financial status, parental studies and occupation, residential geographical positioning compared to the big cities and centers with available gastroenterology and endoscopic services, means of transportation and parental perception of the need for independence of the future young adult. The questionnaire used for data collection is provided as Supplementary Materials.

Later, the questionnaire was evaluated by two separate teams each formed by one pediatric gastroenterology specialist and two gastroenterology and hepatology specialists. Feedback from both panels was synthesized and further assessed by a third team consisting of two professors specializing in pediatric gastroenterology and hepatology.

The two subject matter experts independently reviewed each questionnaire item using a 4-point Likert scale to assess both relevance and clarity (1 = not relevant, 4 = highly relevant). The Item-Level Content Validity Index (I-CVI) was calculated as the proportion of experts who rated each item as either 3 or 4. The Scale-Level CVI using the average method (S-CVI/Ave) was computed as the mean of the I-CVI values across all items, while the universal agreement method (S-CVI/UA) represented the proportion of items for which both experts provided ratings of 3 or 4.

The content validity results were as follows:

- S-CVI/Ave = 0.95;
- Expert 1 Proportion Relevance = 0.96;
- Expert 2 Proportion Relevance = 0.93;
- S-CVI/UA = 0.90.

These values indicate excellent content validity, as they meet or exceed commonly recommended thresholds [16]. It is important to note that only content validity was assessed in this study. No further psychometric validation procedures—such as exploratory or confirmatory factor analysis, construct validity or criterion-related validity—were conducted. This limitation is clearly acknowledged in the manuscript.

### 2.3. Data Collection

After obtaining informed consent, data collection was carried out in November 2020. Legal guardians completed the survey electronically. The questionnaire was self-administered, designed to be completed within approximately 5 min and submitted anonymously. Responses were automatically collected and securely stored for analysis.

### 2.4. Statistical Analysis

All statistical analyses were performed using IBM SPSS Statistics 29.0.2.0 (IBM Corp., Armonk, NY, USA) and Minitab version 22.1 (Minitab LLC, State College, PA, USA) for data visualization, including correlograms (Pearson correlation heatmaps). Quantitative variables were expressed as absolute values and percentages. All statistical tests were two-tailed.

Given the fact that multiple comparisons increase the risk for type I errors, which can lead to false positive results, we applied the Holm–Bonferroni correction to adjust our significance levels, balancing the risk for type I and type II errors. All *p* values discussed in the text are *p* = 0.000, and, by applying the Holm–Bonferroni correction, extremely small and significant values were indicated. Thus, even with Holm–Bonferroni adjustments, these values will remain statistically significant. This correction method was applied according to the procedure proposed by Holm (1979) [17].

## 3. Results

### 3.1. Sociodemographic Characterization

A total of 174 patients and their legal guardians were selected; 124 (71.26%) legal guardians agreed to participate in the study. Sociodemographic data are represented in Table 1.

Responders declared that the majority of teenagers attended school at the time of the investigation (97.5%, *n* = 121).

**Figure 1:**
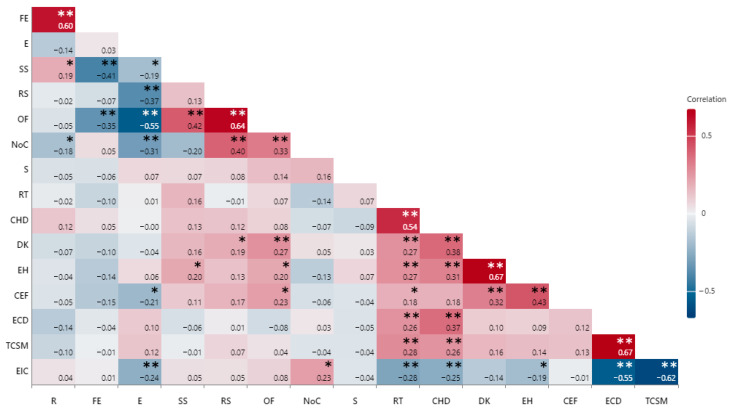
Pearson’s correlation coefficients between sociodemographic and parental perception of adolescents’ transition readiness. The high and low intensity of the colors represent strong and weak relationships (red for positive and blue for negative) between a pair of parameters, respectively. R—respondent, FE—family environment, E—environment (urban/rural), SS—social status, RS—respondent studies, OF—occupational and financial status, NoC—number of children, S—school attendance, RT—perception of transition readiness, CHD—allowing children to make health-related decisions, DK—chronic disease knowledge, EH—encourage healthcare tracking skills, CEF—children express freely, ECD—encouraging children to make decisions on their own, TCSM—trust in children’s self-management skills, EIC—encouragement of independent children’s consultations; * *p* < 0.05, ** *p* < 0.01.

We identified a strong positive relationship between the respondents’ relationship with the patients and the characteristics of the family environment (0.604; *p* = 0.000). Pearson’s correlation coefficients between the sociodemographic and parental perception of adolescents’ transition readiness are synthetized in Figure 1.

The financial status and occupations of guardians were assessed to evaluate access to treatment beyond the age of 18; 20.2% (*n* = 25) reported that their primary income sources were children’s state allowance and social financial aid and 79.8% indicated that at least one parent was employed with 68.5% of participants earning less than EUR 1000 per month.

In completion of the social profile of the target population for specialized translational training, a strong negative relationship was found between parents’ occupation and financial status and the living environment (−0.553; *p* = 0.000).

For the demographic analysis, participants reported the distance from their homes to the pediatric gastroenterologists’ offices. Specifically, 6 (4.8%) lived in the same city, 4 (3.2%) lived 10–15 km away, 15 (12.1%) lived 15–30 km away, 36 (29%) lived 30–50 km away, 40 (32.3%) lived 50–70 km away and 23 (18.5%) lived more than 70 km away. Regarding transportation methods, 73.9% (*n* = 90) primarily used public transportation, while 26.2% (*n* = 32) used personal cars. A statistically significant positive correlation was found between transportation mode and residential location (0.476; *p* = 0.000).

### 3.2. Parental Perception of Adolescents’ Transition Readiness

In the legal guardians’ opinion, 60.5% (*n* = 75) of adolescents possessed detailed knowledge regarding their disease and its management. Responders declared that in 10.5% (*n* = 13) of cases, they did not allow their children to freely express their feelings, thoughts and wishes when making new decisions. The other 89.5% (*n* = 111) declared including their children in this process. Teenagers were encouraged to keep a record of their health and the treatments they were following in 74% (*n* = 91) of cases. The statistical analysis identified a positive significant relationship between responders allowing their children to express freely and encouraging them to keep records of their health and medications (0.430; *p* = 0.000). Similarly, a strong positive correlation between participants encouraging their children to keep healthcare records and their perception of the children’s chronic disease knowledge (0.67; *p* = 0.000) was identified.

Parents and surveyed guardians appreciated that 21% (*n* = 26) of the teenagers in their care could make decisions on their own regarding their health and the necessary medication. Faith in children’s self-management abilities was expressed by 33.87% (*n* = 42) of responders; however, only 32.26% (*n* = 40) of parents encouraged their teenage children to make decisions on their own regarding their health. Independent behaviors such as alone consultations were encouraged by 16.93% (*n* = 21) of responders. We identified a positive statistically significant relationship between parents’ trust in their children’s self-management skills and them encouraging their children to make decisions on their own (0.673; *p* = 0.000).

Responders declared that, in their opinion, 21.8% of adolescents (*n* = 27) were prepared for the transition, while 78.2% (*n* = 97) stated they did not consider their children to be ready for the process. A noteworthy positive correlation was found between parents’ perception of the child being ready to make health-related decisions on their own and parents’ perception of their children’s preparedness for transition to adult medicine (0.544; *p* = 0.000).

**Figure 2:**
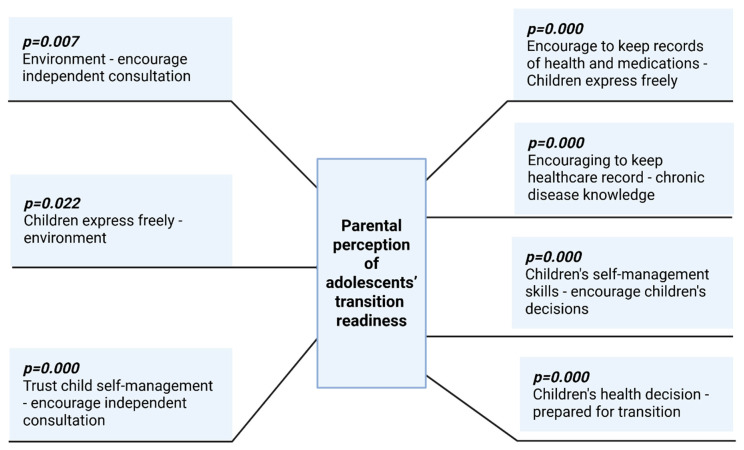
Parental perception of adolescents’ transition readiness.

Figure 2 shows a diagram illustrating parents’ perceptions of adolescent readiness for the transition to independent healthcare. The diagram is organized in the form of a tree with branches detailing various aspects and concerns related to this transition; on the right side are the positive relationships (identified through Pearson’s correlation) and the left side the negatives.

### 3.3. Parental Needs and Fears Regarding the Transition Process

The following section of the survey assessed perceived worries and fears, expectations and needs of responders.

A substantial proportion of participants (89.5%; *n* = 111) expressed concerns regarding medical insurance and the costs associated with disease management after the age of 18. Concerns about potential therapeutic breaks during transition were reported by 99.2% (*n* = 123). When asked about their awareness of available medical care after the age of 18, 80.3% (*n* = 98) were unsure, while only 19.7% (*n* = 24) felt informed. Additionally, 73.4% (*n* = 91) were uncertain about managing disease exacerbations without an adult doctor, with 97.6% (*n* = 121) willing to contact their pediatric gastroenterologist in such cases. Furthermore, 75.8% (*n* = 94) desired continuous guidance from the pediatric gastroenterologist regarding disease management. A significant positive correlation was identified between fears of acute disease episodes after the age of 18 and lack of knowledge about where to seek care (r = 0.63, *p* = 0.000).

**Figure 3:**
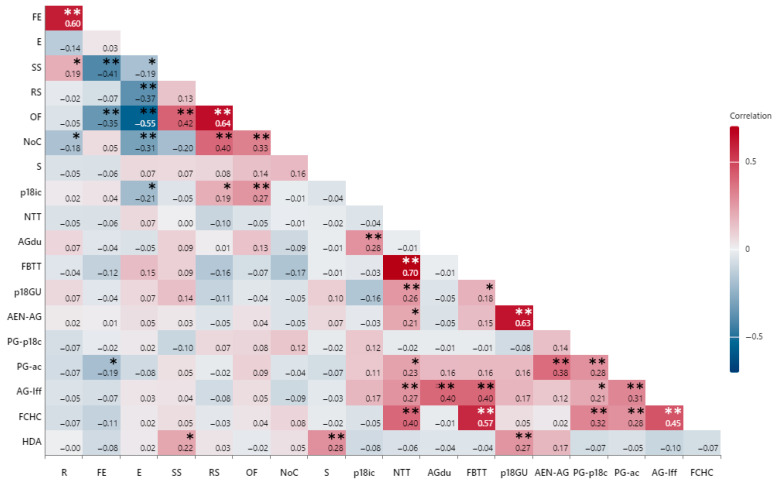
Pearson’s correlation coefficients between sociodemographic data and parental needs and fears regarding the transition process. The high and low intensity of color represent strong and weak relationships (red for positive and blue for negative) between a pair of parameters, respectively. R—respondent, FE—family environment, E—environment (urban/rural), SS—social status, RS—respondent studies, OF—occupational and financial status, NoC—number of children, S—school attendance, p18ic—post-18-years-old insurance costs, NTT—need for transition training, AG-du—adult gastroenterologist discussion useful, FBTT—fear of therapeutic breaks during transition, p18GU—post-18 guidance unknown, AEN-AG—fear of an acute episode after turning 18 in the absence of an adult gastroenterologist, PG-p18c—pediatric gastroenterologist post-18 call, PG-ac—PG advice continues, AG-lff—AG less family-focused, FCHC—fear for child’s impropriate health-related choices, HDA—children’s healthcare decisions creating parental anxiety; * *p* < 0.05, ** *p* < 0.01.

Pearson’s correlation coefficients between sociodemographic data and identified parental needs and fears regarding the transition process are synthetized in Figure 3.

A significant proportion of participants (95.1%; *n* = 116) expressed concerns that adult physicians would be more patient-centered and less family-oriented. Anxiety regarding their children making independent healthcare decisions was reported by 83.06% of respondents. Additionally, 97.6% (*n* = 120) feared their children would make inappropriate health-related choices, and all participants expressed a desire to remain actively involved in disease management after the age of 18. Statistical analysis revealed a significant positive correlation between parents’ fears of inappropriate health-related choices, fears of therapeutic breaks during transition (r = 0.573, *p* = 0.000) and perceptions of adult physicians being less family-centered (r = 0.453, *p* = 0.000).

Regarding the necessity of a structured transition process, 98.4% (*n* = 122) of participants acknowledged its importance. There was also a strong positive correlation between fears of therapeutic breaks and the perceived need for transition training (r = 0.704, *p* = 0.000). Furthermore, 99.2% felt that consultations with an adult gastroenterologist would be beneficial.

**Figure 4:**
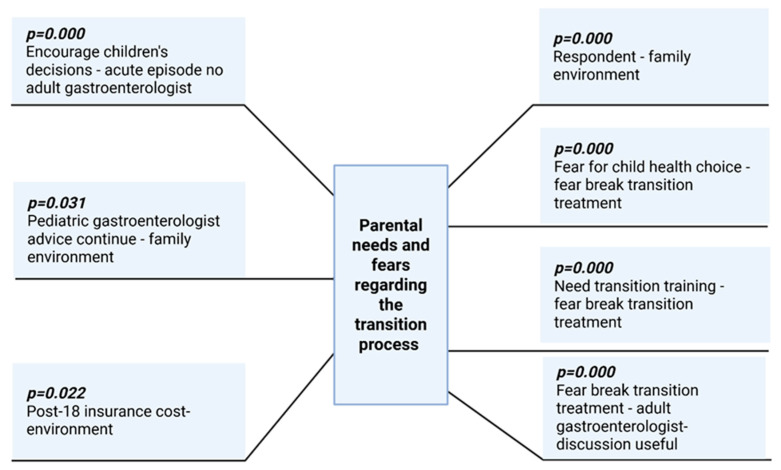
Synthesis of parents’ needs and fears regarding the transition process of their children from pediatricians to adult physicians.

For a clearer and better understanding of the data, Figure 4 presents a synthesis of parents’ needs and fears regarding the transition process of gastroenterology care for their children from pediatricians to adult physicians. On the right side of the figure, the significant positive correlations (identified through Pearson’s correlation) are presented and on the left side, the negative ones, with the level of significance also included.

We performed linear regression analysis on variables exhibiting statistically significant correlations to evaluate the strength and direction of their relationships. The regression results provide an overview of how these variables are associated, with coefficients indicating the magnitude and direction of the potential effects (Table 2).

## 4. Discussion

Long-term diseases among adolescents represent a growing public health concern. During this period, parents continue to actively and independently manage the teenagers’ chronic conditions (such as maintaining communications with the doctors, scheduling appointments, obtaining prescriptions), which creates a relation of dependence between the future young adults and their parents [18]. Parents often share concerns with pediatricians about ensuring continuity of care and safeguarding the well-being of their children. They experience heightened anxiety during the transition period, as they struggle to balance supporting their child’s independence with the fear of reduced oversight and potential health risks [19].

After successfully completing the process of transition into adult healthcare, an important number of patients continue to live with their parents and to consult with them on healthcare matters [20]. This cultural and institutional context where adolescents are encouraged toward autonomy is still held within protective structures and resonates with parents’ ambivalence about stepping back [21]. Authors report that routine healthcare utilization tends to decline after transfer and emergency room visits followed by hospital admissions have the tendency to increase [20,22].

The presence of a chronic disease during childhood has been correlated with poor quality of life for both patients and their caretakers, with a negative impact on the family function. As autonomy develops, parental concern should tend to shift from direct management to ensuring a safe and effective transition to self-care—a process echoed in broader pediatric chronic illness contexts [23].

Another concern is related to the well-being of the healthy siblings who tend to experience high levels of anxiety and depression [24]. Families with multiple children, especially those with more than three, may face significant challenges in allocating time, attention and resources effectively. The needs of the ill child can dominate family resources, potentially neglecting the needs of healthy siblings. Implementing family-centered care programs that offer psychological and emotional support to all family members, including healthy siblings, can mitigate some of the adverse effects.

In Romania, divorce rates have fluctuated slightly over time, ranging from 1.0 to 1.75 divorces per 1000 inhabitants between 1990 and 2021. Although there are minor annual variations, the overall trend has remained relatively stable [25]. Given the relatively high and stable divorce rate in the post-communist period, we considered parental marital status as a relevant sociodemographic variable in our study. Differences in family structure—such as children raised in single-parent households or by separated/divorced caregivers—may influence the degree of parental involvement, perceived readiness for transition and levels of concern regarding the adolescent’s ability to manage their chronic condition independently.

Parents’ anxiety is nurtured not only by the transition itself but also by the accumulation of stress and emotions of raising and caring for a child who, in several cases, needed a large battery of tests and hospitalizations, from infancy (for example, cow’s milk protein allergy is associated with later onset of Crohn’s disease) [26,27].

Previous studies suggest that parents of children with chronic conditions tend to be over-involved in teenagers’ lives and to be reluctant to let go of their children, and the children are more likely to become dependent on their parents and preserve poor autonomy [28]. Similar behaviors have been identified in our study, with all subjects expressing the desire to continue to be actively involved in the management of future adults’ disease.

The results in our study indicate that there is a relationship between parents allowing their children to express themselves freely and parents encouraging adolescents to keep records of their health (0.430; *p* = 0.000). Fostering this relationship may encourage teenagers to take ownership of their health and well-being and may nurture a sense of autonomy and responsibility, which are essential for effective self-management, particularly in the context of chronic conditions.

A socio-economic analysis of the North East region of Romania from 2014 to 2020 indicates that the majority of the population has high school or vocational education (50.2%), followed by those with secondary and primary education (34.5%), and only 12.6% have higher education [29]. Data from the Ministry of Labor, Family and Social Protection’s “Report on Social Inclusion in Romania 2010” reveal that this region has the highest proportion of the poor population in Romania (25.9%), with the absolute poverty rate rising from 6.1% in 2009 to 7.7% in 2010 [30].

Our study highlights a negative correlation between parents’ occupation, financial status and their living environment (r = −0.553, *p* < 0.01), suggesting better access to employment, education and healthcare for urban families. These results align with existing data on economic disparities between urban and rural areas in Romania [31]. Therefore, systematic transition programs should prioritize families in rural areas facing financial difficulties, as children from unstable environments are more likely to experience disengagement and healthcare decline [32].

In our study we have identified a moderate negative relationship between parental social status and family environment stability (−0.41; *p* = 0.000), which can help design targeted interventions and build support systems for children from divorced or unmarried families to help improve their family environment, especially since it appears that single-parent households or households where parents are divorced might face more financial challenges, leading to higher levels of stress and conflict [33]. This finding represents an important factor to consider when assessing the well-being of children and designing interventions for a successful transition process. One strategy could take into consideration the implication of various associations and institutions that provide financial help to chronically ill adolescents and young adults with special healthcare needs (such as the Romanian Association for Gluten Intolerance and Coeliac disease) [34,35].

Data from one systematic review show the complicated and sometimes conflicted aspects that parents experience during their children’s transition from pediatric to adult care, referring strictly to children with chronic conditions. This review identified an inner personal conflict that was described as being cross-pressured, referring to an intrapersonal conflict that arises when the motives influencing a decision are incompatible [18,36].

This concept illustrates parents’ pressure and obligations to meet both the children’s and doctors’ expectations of support during the process of transition. Parents find themselves dealing with two situations: facing internal and external social pressure and having to care for a young person transitioning both to a new healthcare system and to adulthood. Research shows that parents are striving for consistency between their feelings and their actions [18].

In a study analyzing parents and patients with congenital heart diseases, parents expressively underlined the importance of their involvement in the transition process in order to be able to gradually hand over responsibility to the future young adult. Parents considered it important to establish contact with the adult’s medical team before the actual transfer and to ensure that sensitive disease-related information impacting patients’ quality of life would be provided to the future medical team [37,38]. The results of our analysis align with published data regarding the wishes of parents who care for adolescents on the verge of transition; in our study, 98.4% of parents considered transition preparations necessary and 99.2% required a consultation or a discussion with an adult specialist.

In our study we identified a strong positive correlation between responders’ perception of the child being ready to make healthcare decisions on their own and their perception of the children’s preparedness for transition to adult medicine (95% CI, *p* = 0.000), suggesting that parents who foster a sense of autonomy in their children regarding healthcare decisions are more likely to empower them to navigate their healthcare journey more independently, thus facilitating a smoother transition to adult healthcare systems. Understanding and acknowledging these results may empower healthcare professionals to provide comprehensive support, ensuring both parents and children navigate the transition process with confidence. While our cross-sectional data do not allow for causal inferences, this correlation suggests a potential relationship worth exploring in future longitudinal or experimental studies.

The relationship between participants encouraging children to keep records on their healthcare and medication and their perception of the children’s knowledge of the chronic disease (0.67; *p* = 0.000) may indicate that encouraging children to take an active role in managing their health empowers them to become more knowledgeable and involved in their care, leading to better health outcomes and a greater sense of autonomy.

Transition specialists may support such behavior by fostering open communication between parents and children that might allow children to ask questions, express concerns and gain a deeper understanding of their condition, favoring adolescents’ self-efficacy [39].

The term self-efficacy was initially conceptualized as a way to understand the cognitive and motivational processes underlying a particular behavior. Self-efficacy represents an umbrella term referring to the concept of perceived control [40].

Nowadays, self-efficacy refers to an individual’s judgment regarding their own ability to organize and execute specific actions [41]. In this particular context, self-efficacy reflects the individual perception of being able to live successfully with a chronic digestive disease and to successfully engage in self-care behaviors.

The results of our study suggest that parents who encourage self-efficacy behaviors are less likely to fear their children experiencing a severe health episode without medical supervision after turning 18 (95% CI, *p* = 0.000), emphasizing the importance of promoting autonomy in healthcare decision-making and effective transition planning. Encouraging children to make health-related decisions on their own empowers them to take ownership of their health and medical care. This can help ease parents’ fears about their children’s ability to manage their health independently after turning 18 [42].

Our responders declared that 95.1% of them were worried that the adult doctor would be more patient-centered and less family-oriented. In addition, feelings of anxiety were declared by 83.06% of them related to the simple thought that their children would soon have to make decisions on their own regarding healthcare aspects. In a study analyzing parents’ and health professionals’ experiences throughout the transition process, the authors emphasized the fact that legal guardians worried that their children would not receive proper care in the adult-oriented healthcare system, especially related to personalized care and close follow-up. Adult doctors, on the other hand, despite recognizing the need for a closer relationship with their patients, declared experiencing significant external pressure to meet the expectations of both patients and their families. Another major parental concern was related to adult physicians’ intention to change the treatment regimen immediately upon transfer, which increased parents’ anxiety [43].

An additional contextual factor that may influence parental concerns during the transition process is the deficit of healthcare personnel, particularly in the field of pediatric gastroenterology in Romania. The ongoing exodus of medical professionals, a phenomenon well-documented over the past two decades, has resulted in limited access to specialized care, especially in rural and underserved regions. Additionally, the pediatric gastroenterology specialty was only created in Romania 2017 and the first specialists work mainly in the metropolitan areas [44]. This scarcity may contribute to heightened parental anxiety regarding the continuity and quality of care their children will receive after transition. Although our study did not specifically quantify this concern, it is plausible that the perceived or real shortage of trained adult gastroenterologists—particularly those experienced in managing complex pediatric-onset conditions—amplifies caregivers’ fears surrounding therapeutic breaks, miscommunication or suboptimal disease management in the adult system. Future studies should explore this structural issue more explicitly, as it represents a significant barrier to successful transition and long-term outcomes for adolescents with chronic digestive disorders.

Parental excessive involvement in adolescents’ medical care has been noticed by multiple authors, including establishing and accompanying them to medical appointments, providing children’s medical history, answering and asking questions on their behalf during clinic visits, keeping track and picking up medications [41]. In our research, 99.2% of legal guardians worried about possible therapeutic breaks during the transition and 97.6% of them feared that their children would not make adequate choices regarding their health. Surprisingly, all of our participants claimed they wanted to continue to actively participate in the medical management of their children’s chronic disease after the child had turned 18. Similarly, in one study, parents also reported they kept being involved in their children’s chronic disease management even after the young adult had left the house; they declared regularly checking in with them in order to verify that they were following the doctor’s prescription, constantly calling to remind them about medical appointments, which in some cases were made by the parents, and even driving to their home to accompany them to clinic visits [42].

In our study we identified that parents who express a greater fear of therapeutic breaks during transition are also more likely to perceive the need for transition training as essential (0.704; *p* = 0.000), reflecting their awareness of potential challenges and risks associated with the transition process. This finding highlights the importance of providing adequate and proactive support, education, resources and training programs tailored to facilitate a smooth transition for both parents and children. Access to systematic transition programs can help alleviate parents’ concerns and equip them with the knowledge and skills necessary to effectively navigate the transition.

The positive relationships between parents’ fears about their children’s health-related choices and therapeutic breaks during transition (0.573; *p* = 0.000) and their perceptions of adult physicians’ patient orientation (0.453; *p* = 0.000) identified in our research highlight the importance of addressing parents’ fears and concerns during the transition from pediatric to adult healthcare. Healthcare providers should engage in open communication with families, provide information about the transition process and address any anxieties or uncertainties parents may have. We believe that consultations with an adult gastroenterologist prior to the transfer, as part of a systematic process, would help address some of these worries.

The results of our research highlight a significant relationship between parental fear and a lack of awareness about support resources as their children transition into adulthood (0.63, *p* = 0.000). In order to properly address and influence this relationship, mentorship programs could be created where parents of older patients can guide those with teenagers.

Brochures and other informative materials may be delivered to patients and their families with sufficient time prior to the transfer, offering them the possibility to analyze available care facilities for adults.

The ongoing advancement of technology and the integration of telemedicine have increasingly shaped transition programs globally. Notable examples include digital tools such as the Mobile Transition Navigator Application (MTNA), MyHealth Passport and TransitionMate—a mobile application developed for Australian adolescents aged 16 years and older who are expected to transition to adult healthcare services within the following 12 months. These platforms aim to enhance transition readiness by promoting self-management, facilitating information transfer and supporting communication between patients and healthcare providers [45,46,47,48,49,50].

Among the proposed strategies to build up autonomy, several experts recommend that parents be separated from their children during medical visits and firm limits should be set on parental involvement [42]. However, legal questions may arise from consulting a minor without their legal guardian, even if they signed the informed consent forms. We tried to identify such practices in the studied population and we found out that 45.15% of parents did not allow their children to be consulted alone and 37.90% declared feeling uncomfortable when this happened.

This study has some limitations that should be mentioned. Firstly, the study design does not allow researchers to generalize the results to all families with teenagers suffering from chronic digestive diseases across the country, as the study sample was limited to a few families from the North East Region of Romania. Secondly, the investigated population consisted of a relatively reduced number of participants. Thirdly, the investigation method was limited to questionnaires with closed questions completed at home, leading the way for possible bias. The relationship between healthcare providers and participants might influence the responses. Parents who have a close, positive relationship with their child’s medical team may provide more favorable responses, potentially influencing the results. Conversely, parents who had negative experiences might respond more critically. Additionally, parents might respond in ways they believe are expected, especially if they think their responses could impact their children’s medical care. We have tried to diminish the potential social desirability bias by ensuring the anonymity of shared data, and by including in the informed consent forms statements that reassure parents that participation in this study would not influence their children’s medical care.

To our knowledge, this is the first Romanian research that studies families with children suffering from chronic digestive diseases who are on the verge of transitioning from the pediatric healthcare system to the adult-oriented one.

## 5. Conclusions

This study characterizes the intrafamilial environment of chronically ill adolescents in North-Eastern Romania, identifying various risk factors affecting their transition to adult healthcare. Non-modifiable risk factors were noted, but the study also highlighted several modifiable factors that could be targeted through early interventions during an organized smooth transition protocol. These interventions include promoting adolescents’ self-management skills by promoting independent consultations, organizing counseling sessions for adolescents during which formal information on the disease and the followed medication would be provided, organizing consultations with an adult specialist before transfer, providing written information on health insurance and outlining care options after the age of 18.

The investigation into the experiences and concerns of parents of chronically ill adolescents transitioning to adult medicine underscores the critical need for comprehensive, targeted support strategies. As we look ahead, the path forward must include robust longitudinal studies to deepen our understanding of the transition. By identifying and addressing potentially modifiable risk factors, we can develop more effective interventions and we can allocate resources that ensure a smoother, more successful transition for these adolescents. Future research should focus on long-term outcomes and the efficacy of various support mechanisms, ultimately guiding healthcare policies and practices toward a more seamless transition to adult care for chronically ill adolescents.

## Figures and Tables

**Table 1:** Sociodemographic characterization of the studied population.

				Responders	
				%	*n*
**Environment**	Urban			26.5%	33
	Rural			73.4%	91
**Responder’s relationship to patient**	Mother			81.5%	101
	Father			9.7%	12
	Grandparent			2.4%	3
	Foster parent			6.5%	8
**Family environment stability**	Living with both parents			55.6%	69
	Living with one parent		Mother	30.6%	38
			Father	4.8%	4
	Living in foster care homes			6.5%	8
	Living with grandparents			2.5%	3
**Parental legal status**	Married			45.2%	56
	Divorced			27.4%	34
	Cohabitating			19.4%	24
	One deceased parent			8%	10
**Family components**	One child			6.5%	8
	Two children			25.8%	32
	Three children			28.2%	35
	Four/five children			28.2%	35
	More than five children			11.3%	14
**Legal guardian‘s education level**	Primary school			0.8%	1
	Secondary school			1.6%	2
	Highschool	Graduated		30.6%	38
		Not graduated		28.8%	37
	Higher education (school of arts)			25.8%	32
	University			11.3%	14

**Table 2:** Linear regression analysis of the significant correlations.

Variable 1	Variable 2	R Square	*p*-Value	Observations
**Living environment**	Encouragement of independent consultation	0.7000901	*p* = 0.000	124
**Living environment**	Allowing children to express themselves freely	0.79507		
**Trust in children’s self-management abilities**	Encouragement of independent consultation	0.66743		
**Encouragement to keep records of their health and medication**	Allowing children to express themselves freely	0.90129 *		
**Encouragement to keep records of their health and medication**	Chronic disease knowledge	0.929443 *		
**Trust in children’s self-management abilities**	Encouragement of children’s decision	0.952386 *		
**Encouragement of children’s decision**	Perceived level of preparedness for the transition	0.954914 *		
**Encouragement of children’s decision**	Fear of an acute episode in the absence of an adult gastroenterologist	0.751372		
**Desire to receive continuous advice from the pediatric gastroenterologist**	Family environment	0.69458		
**Healthcare insurance costs after the age of 18**	Living environment	0.79507		
**Respondent**	Family environment	0.824273		
**Fear for children‘s healthcare-related choices**	Fear of therapeutic breaks during transition	0.985199 *		
**Need transition training**	Fear of therapeutic breaks during transition	0.992368 *		
**Fear of therapeutic breaks during transition**	Usefulness of a discussion with an adult gastroenterologist	0.984314 *		

R square cut-off: 0.9; significant results are marked with *.

## Data Availability

The data presented in this study are available on request from the corresponding author due to the large amount of data and privacy concerns.

## Supplementary Materials

The following supporting information can be downloaded at: https://www.mdpi.com/article/10.3390/medicina61081338/s1, Questionnaire for Legal Guardians.
