# Supplementary material for: The Journey to Autonomy: Understanding Parental Concerns During the Transition of Children with Chronic Digestive Disorders
Source: Medicina (Kaunas). 2025 Jul 24;61(8):1338. doi: 10.3390/medicina61081338 (PMC12387450; doi:10.3390/medicina61081338)
Supplement: Supplementary file 1 [file medicina-61-01338-s001.zip › medicina-3761892-supplementary.pdf]

QUESTIONNAIRE FOR LEGAL GUARDIANS

Instructions: Please check the box that best describes your skill level in the following areas that assess the transition process to adult medicine. There is no right or wrong answer and your answers will be transmitted anonymously.

1. Who are you in relation to the patient?
- Mother
- Father
- Foster parent
- Grandparent

2. Who does the patient live with?
- Their mother
- Their father
- Both their parents
- With the foster parent
- Their grandparents

3. In what environment do you live?
- Urban
- Rural

4. What is the parents' civil status?
- Married
- Divorced
- Cohabitating
- One deceased parent

5. What are parents' level of education?
- Primary school (4th grade)
- Secondary school (8th grade)
- Highschool- started but not graduated from it
- Highschool graduated
- Higher education (school of arts)
- University

6. Income per family/month:
- Mainly children's state allowances
- Social assistance aid and children's state allowances
- Wages, less than 2000 lei (400 euro)
- Wages, between 2000-3000 lei (400-600 euro)
- Wages, 3000-5000 lei (600-1000 euro)

- Wages, 5000-8000 lei (1000-1600 euro)
- Wages, over 8000 lei (1600 euro).

7. What is the distance from your home to the city of Iasi:
- 0 /km (I live in Iași)
- 10-15 Km
- 15-30 Km
- 30-50 Km
- 50-70 Km
- Over 70 Km

8. What means of transport do you use to get to the hospital?
- Public transport (bus, tram, train)
- Personal car

9. How many children does your family have?
- One child
- Two children
- Three children
- Four or five children
- More than five children

10. Does your son/daughter go to school?
- Yes
- No

Answer Yes or No to the following questions/mark True) or False to the following statements:

11. Do you think that your son/ daughter is ready for the transition to adult medicine?
- **Yes**
- **No**

12. Do you think that your son/daughter can make decisions on their own regarding their health condition and necessary medication?
- **Yes**
- **No**

13. My child knows details regarding their disease and the followed medication.
- **True**
- **False**

14. I encourage my child to keep a record of their health and the followed medication.
- **True**
- **False**

15. I allow my child to freely express their thoughts, feelings and wishes when new decisions are to be made.
- **True**

○ **False**
**16. I am wondering about the type of medical insurance my child can have after the age of 18 or the costs involved in managing their illness.**<br>○ **True**<br>○ **False**
**17. Do you think it would be necessary to have a training before the transfer to adult medicine?**<br>○ **Yes**<br>○ **No**
**18. Do you think it would be useful to discuss with an adults' gastroenterologist?**<br>○ **Yes**<br>○ **No**
**19. I fear that during the transition process there will be therapeutic breaks.**<br>○ **True**<br>○ **False**
**20. I don't know who/where to turn to after my child turns 18.**<br>○ **True**<br>○ **False**
**21. I do not know what to do if, after the age of 18, my child has an acute episode of the disease and does not yet have an adult-oriented attending physician.**<br>○ **True**<br>○ **False**
**22. If, after the age of 18, my child has an acute episode of the disease and does not yet have an adult attending physician, I will call/contact the pediatric gastroenterologist.**<br>○ **True**<br>○ **False**
**23. I wish to continue to consult with the pediatric gastroenterologist about decisions related to the management of my child's disease.**<br>○ **True**<br>○ **False**
**24. I fear that the adult specialist would be more patient-centered and less family-oriented.**<br>○ **True**<br>○ **False**
**25. I fear that my son/daughter would make inappropriate decisions regarding their health.**

○ **True**<br>○ **False**
**26. I encourage my child to make decisions regarding their health on their own.**<br>○ **True**<br>○ **False**
**27. I get anxious when I think about my son/daughter having to make their own healthcare decisions.**<br>○ **True**<br>○ **False**
**28. I would like to actively participate in process of decision making related to the management of my child's illness (after the age of 18).**<br>○ **True**<br>○ **False**
**29. I trust my child's abilities of self-management of their chronic disease.**<br>○ **True**<br>○ **False**
**30. Do you encourage you minor child to be independently consulted in your absence?**<br>○ **I do not agree to be consulted in my absence**<br>○ **I feel uncomfortable when my child is consulted in my absence**<br>○ **Yes, I agree and I encourage this habit.**
